# Identification and characterization of aging/senescence-induced genes in osteosarcoma and predicting clinical prognosis

**DOI:** 10.3389/fimmu.2022.997765

**Published:** 2022-10-05

**Authors:** Yigang Lv, Liyuan Wu, Huan Jian, Chi Zhang, Yongfu Lou, Yi Kang, Mengfan Hou, Zhen Li, Xueying Li, Baofa Sun, Hengxing Zhou

**Affiliations:** ^1^ Department of Orthopaedics, Tianjin Medical University General Hospital, International Science and Technology Cooperation Base of Spinal Cord Injury, Tianjin Key Laboratory of Spine and Spinal Cord, Tianjin, China; ^2^ State Key Laboratory of Molecular Oncology, National Cancer Center/National Clinical Research Center for Cancer/Cancer Hospital, Chinese Academy of Medical Sciences and Peking Union Medical College, Beijing, China; ^3^ Department of Orthopaedics, Shandong University Centre for Orthopaedics, Qilu Hospital, Cheeloo College of Medicine, Shandong University, Jinan, China; ^4^ Key Laboratory of Immuno Microenvironment and Disease of the Educational Ministry of China, Department of Immunology, Tianjin Medical University, Tianjin, China; ^5^ Shandong University Centre for Orthopaedics, Advanced Medical Research Institute, Cheeloo College of Medicine, Shandong University, Jinan, China; ^6^ Department of Zoology, College of Life Science, Nankai University, Tianjin, China

**Keywords:** aging/senescence-induced genes, osteosarcoma, bioinformatic analyses, anticancer immune cycle, immunotherapy and chemotherapy

## Abstract

**Background:**

Aging is an influential risk factor for progression of both degenerative and oncological diseases of the bone. Osteosarcoma, considered the most common primary mesenchymal tumor of the bone, is a worldwide disease with poor 5-year survival. This study investigated the role of aging-/senescence-induced genes (ASIGs) in contributing to osteosarcoma diagnosis, prognosis, and therapeutic agent prediction.

**Methods:**

Therapeutically Applicable Research to Generate Effective Treatments (TARGET), Gene Expression Omnibus (GEO), and The Cancer Genome Atlas (TCGA) were used to collect relevant gene expression and clinical data of osteosarcoma and paracancerous tissues. Patients were clustered by consensus using prognosis-related ASIGs. ssGSEA, ESTIMATE, and TIMER were used to determine the tumor immune microenvironment (TIME) of subgroups. Functional analysis of differentially expressed genes between subgroups, including Gene Ontology (GO), Kyoto Encyclopedia of Genes and Genomes (KEGG), and gene set variation analyses (GSVAs), was performed to clarify functional status. Prognostic risk models were constructed by univariate Cox regression and least absolute shrinkage and selection operator (LASSO) regression. SCISSOR was used to identify relevant cells in osteosarcoma single-cell data for different risk groups. The effect of immunotherapy was predicted based on TIDE scores and chemotherapy drug sensitivity using CTRP and PRISM.

**Results:**

Three molecular subgroups were identified based on prognostic differentially expressed ASIGs. Immunological infiltration levels of the three groups differed significantly. Based on GO and KEGG analyses, differentially expressed genes between the three subgroups mainly relate to immune and aging regulation pathways; GSVA showed substantial variations in multiple Hallmark pathways among the subgroups. The ASIG risk score built based on differentially expressed genes can predict patient survival and immune status. We also developed a nomogram graph to accurately predict prognosis in combination with clinical characteristics. The correlation between the immune activation profile of patients and the risk score is discussed. Through single-cell analysis of the tumor microenvironment, we identified distinct risk-group-associated cells with significant differences in immune signaling pathways. Immunotherapeutic efficacy and chemotherapeutic agent screening were evaluated based on risk score.

**Conclusion:**

Aging-related prognostic genes can distinguish osteosarcoma molecular subgroups. Our novel aging-associated gene signature risk score can be used to predict the osteosarcoma immune landscape and prognosis. Moreover, the risk score correlates with the TIME and provides a reference for immunotherapy and chemotherapy in terms of osteosarcoma.

## Introduction

Osteosarcoma is considered the most common primary solid malignant tumor of bone and is caused by malignant mesenchymal cells producing osteoid or immature bone (1). In the general population, the incidence of osteosarcoma is 2–3 per million per year, and this disease exhibits a predilection for 0–14 years and over 60 years. Men are 1.4 times as likely as women to be afflicted (2). The etiology of osteosarcoma remains unknown in most individuals. The connection of osteosarcoma with the pubertal growth spurt age and maximal growth locations shows the involvement of fast bone proliferation. Additionally, radiation causes a small percentage of osteosarcomas. Alkylating agents may also play some role in osteosarcoma development. Osteosarcoma is more common in a variety of well-defined hereditary illnesses linked to germline mutations of tumor-suppressor genes (TSGs), such as hereditary retinoblastoma (1) and Li–Fraumeni cancer family syndrome (3), which are associated with an increased incidence of osteosarcoma. The pathophysiology of osteosarcoma has not been clearly elucidated, but many studies have shown that its pathogenesis is closely related to genetic and hereditary factors, and most of the current main treatment modalities require drug chemotherapy in addition to surgery. Based on the need to elucidate the pathogenesis and find more effective chemotherapeutic agents, the screening and identification of osteosarcoma biomarkers have become a hot topic of research.

Cellular senescence is characterized by the accumulation of senescence-associated galactosidase glycosidase (SA-β-gal) and the expression of the senescence-associated secretory phenotype (SASP). Several variables, including oxidative stress and DNA damage, have been associated with cellular senescence (4, 5). The cellular senescence process is mediated by two well-known mechanisms, namely, the p53/p21 and Rb1/p16 pathways; however, cells can also be regulated by pathways that are not dependent on p53 (6). In addition, thioredoxin-interacting protein (TXNIP) can act as another key regulator of cellular senescence (7). Cellular senescence is connected to a range of disorders, and the role of cellular senescence in tumor suppression has become a hot topic of research in recent years in the field of cancer prevention and treatment (8). Cellular senescence has an influential function in different stages of oncogenesis, establishment, and escape (9). A previous study showed that cancer-associated fibroblast (CAF) senescence is closely associated with cancer metastasis (10). Due to the surrounding inflammatory milieu, CAFs in ascites undergo cellular senescence with the progression of gastric cancer to the peritoneum. Furthermore, senescent CAFs continue to emit senescence-associated proteins, which promote tumor growth by activating genes that produce senescence-associated proteins and thereby induce spreading of the cancer to the peritoneum at a much faster pace. According to other studies, aging boosts tumor invasion and recurrence (11).

Bioinformatics methods have recently been used to predict disease target genes and analyze their possible molecular mechanisms and can thus provide more feasible ideas and protocols for subsequent trials. The goals of related studies are to obtain an understanding of the disease pathogenesis and investigate new target drugs, particularly with the development of gene chips and high-throughput sequencing technologies. Researchers in the field of cancer are interested in using aging-/senescence-induced genes (ASIGs) as diagnostic or prognostic molecular biomarkers (12, 13). In contrast, the prognostic roles of ASIGs and their biological functions in osteosarcoma are unknown. Furthermore, an ASIG signature that can reliably predict overall survival (OS)-related survival has not been identified. The goal of this study was to determine whether these ASIGs are linked to osteosarcoma using mRNA expression and clinical data from public sources. We also created and validated a predictive multigene signature.

## Materials and methods

### Data collection

The Genome Data Commons data portal (https://portal.gdc.cancer.gov/) provided us with mRNA expression data from osteosarcoma patients. The complete clinical information of the patients was downloaded from the Therapeutically Applicable Research to Generate Effective Treatments (TARGET) database (https://ocg.cancer.gov/programs/target). The mRNA expression data and clinical characteristics of osteosarcoma samples in GSE21257, the mRNA expression profiles of osteosarcoma and adjacent tissues in GSE99671, and the single-cell RNA sequencing (scRNA-seq) data of osteosarcoma in GSE152048 were obtained from the NCBI Gene Expression Omnibus (https://www.ncbi.nlm.nih.gov/geo/). For the external public cohort, we obtained mRNA expression data for 265 SARC tumor samples from The Cancer Genome Atlas (TCGA) (https://portal.gdc.cancer.gov/). All of the clinical data used in this study can be found in **Supplementary Table S1**. **Supplementary Table S2** contains the demographic information and clinical characteristics of the training and validation cohorts.

We integrated the MSigDB gene sets to establish the expression patterns related to aging. Specifically, the following gene sets were previously established experimentally: GOBP cell aging (M14701), Tang senescence Tp53 targets up (M11850), WP TCA cycle in senescence (M40058), WP senescence and autophagy in cancer (M39619), GOBP regulating cellular senescence (M16568), GOBP positively regulating cellular aging (M24705), and GOBP replicative senescence (M14683) (14). These genes are known as ASIGs because they are upregulated during cellular senescence; they are all listed in **Supplementary Table S3**.

### scRNA-Seq data analysis

Gene expression data for individual samples were analyzed using Read10×() in the Seurat package (v4.1.1) of R software (v4.2.0). Low-quality cells with ≤300 detected genes or ≥10% mitochondrial genes were removed and normalized by NormalizeData(), and the top 3,000 highly variable genes were subsequently identified by FindVariableFeatures(). Batch effects were removed from all samples using the Harmony package (v1.0) (15). K-nearest neighbors were calculated using Harmony-corrected data, and a shared nearest neighbor (SNN) plot was created. A clustering algorithm was then used to find the cell clusters. Using the unified manifold approximation and project (UMAP) dimensionality reduction technique, the identified clusters were visualized on a 2D map. To identify the cell clusters, we first used the SingleR package (v1.10.0) as an auxiliary tool to identify cells and identified differentially expressed genes (DEGs) with high discriminatory power between groups using the FindAllMarkers() function in Seurat with a Wilcoxon rank sum test with Bonferroni correction. DEGs and well-known cell markers in the literature were used to annotate cell clusters. Details of the cellular biomarkers are provided in **Supplementary Table S4**. Based on the R package SCISSOR (v2.0.0) (16) developed by Sun et al., the key step is to quantify the similarity between single-cell data and bulk data by measuring the Pearson correlation of each pair of cells and bulk samples, then optimize the regression model of the correlation matrix using the sample phenotype, and select similar cells that are important for a given phenotype with high confidence. We obtained relevant cells for high- and low-risk grouping by integrating the expression data of patients in TARGET-OS and the risk grouping information of patients. Calculations were performed with default parameters using the R package infercnv (v1.12.0) with annotated immune cells as a reference, and clusters were reassigned *via* the CNV matrix. The CNV score was calculated by the average of the CNV matrix in each cluster. Using the R package CellChat (v1.1.3) (17), the communication network between cells in the tumor microenvironment (TME) was analyzed and visualized.

### Identification of ASIGs in different cluster subgroups

In order to evaluate the prognosis-related genes among ASIGs in osteosarcoma, a combination of univariate Cox regression analysis and single-gene Kaplan–Meier (KM) analysis was used, and genes satisfying p< 0.05 were screened. The results of KM analysis and univariate Cox regression of the prognosis-related genes in ASIGs are listed in **Supplementary Table S5**. Using the STRING database, the protein–protein interaction (PPI) network was constructed with a confidence of 0.18 for the above prognostic genes and analyzed in Cytoscape software. Differential analysis was performed to determine prognosis-related differentially expressed genes among the ASIGs. The program “ConsensusClusterPlus” was then used to perform 500 iterations of consensus clustering using the “pam” method and Pearson distance based on the expression matrix of these 22 genes.

### TME assessment and immune infiltration analysis

Tracking the Tumor Immune Phenotype (TIP) (http://biocc.hrbmu.edu.cn/TIP/) (18), a web-based analysis platform was used to obtain the activation levels of the seven-step anticancer immune cycle. The stromal status, immune status, and tumor purity were assessed for each sample by the ESTIMATE algorithm (19). This algorithm calculates the stromal and immune score of the tumor tissue based on specific characteristics related to stromal and immune cell infiltration in the tumor tissue to predict the level of infiltrating stromal and immune cells and infer the purity of tumor. The single sample gene set enrichment analysis (ssGSEA) approach was then used to calculate the particular immune cells in the TME. **Supplementary Table S6** lists the genes of immune cells (20) used by the ssGSEA algorithm. For immune infiltration analysis, the TIMER and MCPCOUNTER algorithms were used, and the abundance of APC cells was calculated.

### Functional enrichment analysis

The “ClusterProfiler” R package was used to identify enhanced relevant pathways through Gene Ontology (GO) analysis and Kyoto Encyclopedia of Genes and Genomes (KEGG) analysis. The “GSVA” R software package was used to perform gene set variation analysis (GSVA). The alterations in signal pathways among the three clusters were investigated using the “Hallmark” gene set collected from the Molecular Signature Database.

### Construction of a prognostic ASIG signature

We used the R package “survival” to perform univariate Cox analysis of DEGs to identify ASIGs with prognostic value. We then utilized the “glmnet” R package to perform least absolute shrinkage and selection operator (LASSO) regression of the prognostic genes. Four genes were then discovered and used to create a risk score. The risk score was calculated using the following formula: Risk score = (−0.1913) * EVI2B + (−0.1043) * AIM1 + (−0.1502) * PRKACB + (0.2488) * TCEA3. To define the threshold values, we used cutoff values for receiver operating characteristic (ROC) curves based on the ASIG risk scores and split the patients into two different risk score groups. The survival curves were created by Kaplan–Meier (KM) analysis and the log rank test using the “survival” R package to assess the accuracy of the prediction, and ROC curves for the risk scores were constructed using the “timeROC” R package.

### Osteosarcoma cell lines and cell culture

The osteoblast cell line (hFOB1.19) and osteosarcoma cell lines (Saos-2, U2OS, and HOS) were obtained from the American Type Culture Collection (ATCC, Manassas, VA, USA). The cell lines were cultured in Dulbecco’s modified Eagle’s medium (DMEM) containing 10% fetal bovine serum (FBS) and 1% penicillin/streptomycin. All of the cell lines were grown in an incubator at 37°C with 5% CO_2_.

### RNA extraction and quantitative real-time polymerase chain reaction

Total RNA was extracted from the cell lines using TRIzol reagent. Reverse transcription was performed using the Revertaid First-Strand cDNA Synthesis Kit (Thermo Scientific, Cat. No. k1622). The RNA concentration was adjusted using an UltraSYBR mixture, and the samples were then reacted in a Roche real-time quantitative PCR instrument. The primers and their sequences were as follows:

EVI2B-F, 5’-AAGCAGTCACAGCCTACCTTA-3’; EVI2B-R, 5’-TGAATTGTGTTGGTTGACCCAAA-3’; AIM1-F, 5’-GACAGTGACCACTAAAGTGACC-3’; AIM1-R, 5’-GTGGCAGTGTTGCCTTTGT-3’; PRKACB-F, 5’-CCATGCACGGTTCTATGCAG-3’; PRKACB-R, 5’-GTCTGTGACCTGGATATAGCCTT-3’; TCEA3-F, 5’-AAGAGCACGGACATGAAGTACC-3’; TCEA3-R, 5’-CTCTGCCGTCATCTTGGCTA-3’; GAPDH-F, 5’ -ACAACTTTGGTATCGTGGAAGG-3’ and GAPDH-R, 5’-GCCATCACGCCACAGTTTC-3’.

We used GAPDH as an internal reference and calculated the relative mRNA expression level using the 2^−ΔΔCT^ method. This part of the experiment was repeated three times.

### Prediction of drug sensitivity

The Cancer Therapeutics Response Portal (CTRP, https://portals.broadinstitute.org/ctrp) and the PRISM database (https://depmap.org/portal/prism/) were used to collect drug sensitivity data for cancer cell lines (CCLs). The area under the ROC curve (AUC) value of each drug was calculated for osteosarcoma patients by the ridge regression method using the calcPhenotype function in the R package “pRRophetic” based on CCLs in the CTRP and PRISM databases. Lower AUC values suggested heightened sensitivity to therapy. Subsequently, the difference in AUC values between different risk scoring groups and the correlation with the risk score was evaluated. Connectivity map (CMap) analysis is used to analyze the similar effects of compounds on cell line processing and find drugs to treat diseases (21). Based on the differential genes between the results of tumor and adjacent tissue samples in GSE99671, the CMap score of the drug was obtained using the CLUE (https://clue.io/query). CMap score is a standardized quantity ranging from −100 to 100 and is sorted by “connectivity score.” Negative values indicate the gene expression pattern of a specific perturbation opposite to the disease-specific expression pattern, indicating the potential therapeutic effect of the perturbation on the disease. Drugs with CMap score >90 or< −90 are generally used for further research (22). **Supplementary Table S7** lists the genes utilized for CMap analysis and the CMap scores of all drugs.

### Statistical analysis

In this study, statistical operation and visualization were performed using R 4.1.1 software. Based on the recommended methods, the statistical analyses of different datasets were performed using different packages. All the tests were bilateral, and p<0.05 was considered to indicate statistical significance in all the tests.

## Results

### Identification of three molecular subtypes based on ASIGs

**Figure 1:**
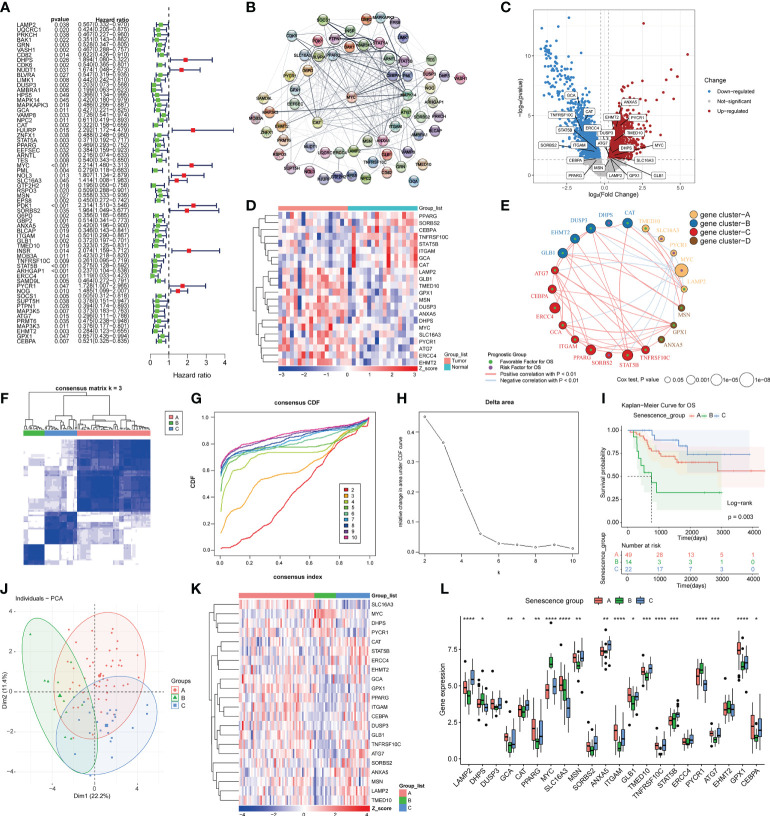
Prognostic gene screening and consensus clustering. **(A)**Univariate Cox regression of ASIGs for the screening of prognosis-related genes. **(B)** Construction of a PPI network of 64 prognosis-related genes using STRING. **(C)** Differential expression analysis of osteosarcoma samples and adjacent samples in GSE99671 (p-value< 0.05 and log2FC > 0.25). **(D)** Heatmap of 22 prognosis-related differences in ASIGs in GSE99671. **(E)** Coexpression network of 22 prognosis-related genes in TARGET-OS (p-value< 0.05 and correlation > 0.3). **(F)** Heatmap of the consensus matrices for k = 3. **(G, H)** Consensus cumulative distribution function (CDF) plot for 22 ASIGs in TARGET-OS. **(I)** Kaplan–Meier curves based on three clusters in the TARGET-OS. **(J)** Principal component analysis (PCA) of the three subgroups. **(K, L)** Heatmap and box plot of 22 prognosisrelated ASIGs in the three subgroups (*p< 0.05, **p< 0.01, ***p< 0.001, ****p< 0.0001).

We performed KM analysis and univariate Cox regression analysis to investigate 64 genes associated with prognosis in the TARGET-OS data (**Figure 1A**). Using STRING, the interaction network of these 64 genes was created and revealed that these 64 genes may interact with one another (**Figure 1B**). We downloaded data from Gene Expression Omnibus (GEO) encompassing samples of cancer tissues and paracancerous tissues to analyze the DEGs between the two tissues. For GSE99671, volcano plots and heatmaps were used to illustrate 22 DEGs among 64 prognosis-related genes (**Figures 1C, D**). Pearson’s correlation coefficient was utilized to build a coexpression network of 22 prognosis-related DEGs, and these 22 genes were confirmed to be coexpressed in bone malignancies, and according to the expression patterns of these genes, these 22 prognosis-related genes can be clustered into four gene clusters (**Figure 1E**). The osteosarcoma patients in the training cohort were clustered into three groups using a consensus clustering method based on the prognostically relevant DEGs identified in GSE99671 and TARGET-OS. With K = 3, 49 of the patients were assigned to cluster A, and 14 and 22 patients were assigned to clusters B and C, respectively (**Figures 1F–H**). Survival was similarly well discriminated between the three subgroups (p = 0.003) (**Figure 1I**). The patients in cluster B had a lower overall survival rate than those in clusters A and C, whereas the patients in cluster C had the highest overall survival rate. Principal component analysis (PCA) clearly distinguished the three subgroups (**Figure 1J**). The expression levels of ASIGs in the three subtypes are shown in a heatmap and visualized using box plots, which revealed considerable variations in expression among the three clusters (**Figures 1K, L**).

### Immune status of three molecular subtypes and functional analysis

**Figure 2:**
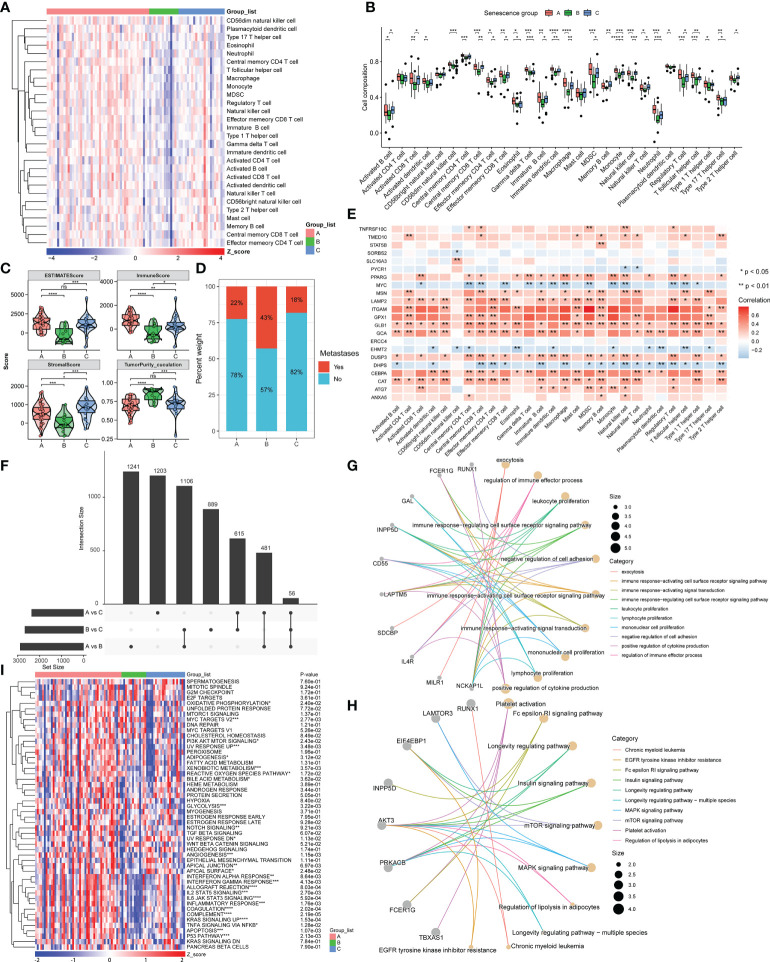
Immune status and functional analysis of DEGs in the three subgroups. **(A, B)** Heatmap and box plot of the statistical analysis of the ssGSEA results. **(C)** Estimate evaluation, immune status, stromal status, and tumor purity calculated for the three clusters using the ESTIMATE algorithm. **(D)** Proportion of patients with metastasis in the three subgroups. **(E)** Correlation between 22 prognosis-related ASIGs and 28 immune cells. **(F)** DEGs in the three subgroups (|log2FC|>0.25, p-value<0.01). **(G, H)** Network diagram constructed from the GO and KEGG analysis of DEGs. **(I)** Heatmap of GSVA results of the hallmark pathway (ns, not significant, *p< 0.05, ** p< 0.01, *** p< 0.001, ****p< 0.0001).

The tumor immune microenvironment (TIME) includes initiation of the anticancer immunity cycle, recruitment of immune cells, expression of immune checkpoint inhibitors (ICIs) and effector genes, and presentation of a T-cell-associated inflammatory signature (TIS). We then explored the immune differences between the three molecular subtypes. The immune cell distribution obtained by the ssGSEA algorithm was significantly different among the three clusters and is presented in a heatmap of 28 immune cells (**Figure 2A**). The scores for 28 immune cells in cluster B were significantly lower than those in clusters A and C, reflecting a worse immune status. By comparing the 28 immune cells between the three clusters, we found that 25 were significantly different. Among the 28 immune cells in cluster A, 7, including immature dendritic cell, monocyte, neutrophil, and type 17 T-helper cell, were the most infiltrated among the three clusters (**Figure 2B**), confirming that the patients in cluster A may exhibit an inflammatory phenotype, corresponding to better immune status. According to the ESTIMATE algorithm, cluster B had the lowest immune score, the lowest stromal score, the highest tumor purity, and the worst overall survival (**Figure 2C**). In contrast, although the immunological score of cluster C was lower than that of cluster A, cluster C had the highest stromal score, a lower percentage of tumor metastasis, and thus the best overall patient survival (**Figure 2D**). We then evaluated the correlations of the 22 prognosis-related ASIGs used for clustering with 28 immune cells and found that 21 of the included genes were significantly associated with at least one immune cell (**Figure 2E**). We then explored the DEGs between the three clusters and performed functional enrichment analysis.

By comparing the DEGs between the three clusters (|DEG_logFC| > 0.25, DEG_p value< 0.01), we found that 56 genes were fully differentially expressed between all three clusters (**Figure 2F**). A GO analysis revealed that the functions of these DEGs were enriched for immune activation, immune cell proliferation, and cell adhesion regulation (**Figure 2G**). A KEGG analysis also reflected several pathways related to cancer and immunity, including the MAPK and mTOR signaling pathways, longevity regulatory pathway, and platelet activation (**Figure 2H**). We then identified hallmark pathways that were significantly different between the three subgroups by GSVA analysis, and these included the P53 pathway, KRAS pathway, glycolysis, and inflammatory response-related pathways. We demonstrated the existence of significant differences among multiple cancer-related pathways based on the three molecular subtypes of ASIGs, which reflected the intrinsic differences in signaling pathways among these three subtypes (**Figure 2I**). In summary, the expression of ASIGs is linked to the immune response and the modulation of cancer-related pathways, which are related to the survival state of patients.

### Development of ASIG subgroup risk scores and validation

**Figure 3:**
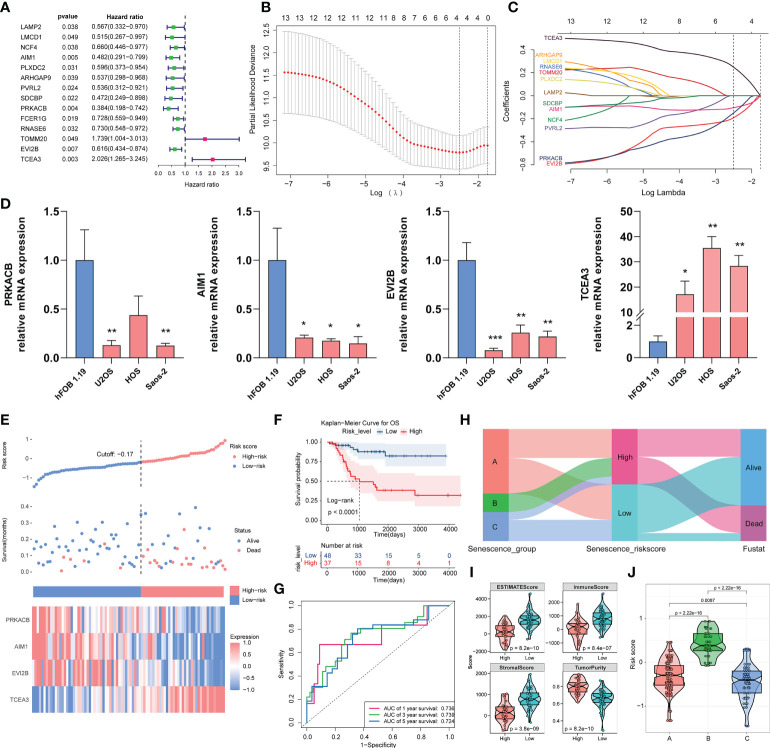
Construction of the risk model with the TARGET-OS cohort and validation with the validation cohort. **(A)** Through univariate Cox regression, the prognostic DEGs in the three clusters were screened. **(B, C)** LASSO regression analysis with optimal lambda. **(D)** Relative mRNA expression of PRKACB, AIM1, EVI2B and TCEA3 in different cell lines by qPCR. (*p < 0.05, **p < 0.01, ***p < 0.001. Not labeled: no statistical significance compared to hFOB 1.19). **(E)** Risk scores constructed from candidate genes, patient survival status, and expression heatmaps of the four candidate genes. **(F)** Survival curve of osteosarcoma patients in different risk groups. **(G)** ROC curve of the risk score model based on four candidate genes. **(H)** Relationship of the risk score grouping and survival status of the patients in the three subgroups. **(I)** ESTIMATE algorithm results for different risk groups. **(J)** Distribution of risk scores in the three subgroups.

The predictive ability of the risk score was assessed. We used Cox univariate regression to screen for 14 prognosis-related genes based on the DEGs in the three subgroups (**Figure 3A**). A LASSO analysis was then performed in the TARGET-OS cohort, and four genes that may be employed in model creation were evaluated to determine their optimal value for building a risk model (**Figures 3B, C**). We also performed quantitative PCR (qPCR) to measure the mRNA expression levels of the four genes in different cell lines. The four osteosarcoma cell lines generally exhibited higher TCEA3 expression and lower PRKACB, AIM1, and EVI2B compared with the osteoblast cell line (hFOB1.19) (**Figure 3D**). The risk score was then calculated using the following equation:


Risk score=(−0.1913)∗EVI2B+(−0.1043)∗AIM1+(−0.1502)∗PRKACB+(0.2488)∗TCEA3

According to the KM analysis, all four genes independently predicted overall survival in osteosarcoma, which demonstrated that they are all prognosis-related genes in osteosarcoma (**Supplementary Figure S1A**). Patients were split into high- and low-risk groups depending on the cutoff value of the risk model. **Figure 3E** depicts the risk scores, patient survival, and inter-cluster prognostic markers. According to the overall survival curves, patients with high-risk scores showed worse survival than those with low-risk scores (p< 0.0001) (**Figure 3F**). We next assessed the predictive ability of the risk model, and a time-correlated ROC analysis revealed that the constructed risk model had strong predictive power at 5 years, with AUCs of 0.736, 0.739, and 0.724 (**Figure 3G**). We also compared the scores of the three clusters of patients using the Sankey diagram and found that the high-risk group contained all the patients in cluster B, nearly half of those in cluster A, and only a few of the patients in cluster C. An analysis of the grouping of risk score versus patient survival status showed that a higher number of the patients in the high-risk score group were at a death state (**Figure 3H**). The ESTIMATE algorithm revealed that the low-risk group had significantly higher stromal scores (p = 3.8e−09), immunological scores (8.4e−07), and ESTIMATE scores (p = 8.2e−10), and lower tumor purity (**Figure 3I**). We found that the risk score of the three clusters corresponded to the survival of each cluster, and significant differences were found among the clusters, implying that our risk ratings were better able to represent the subgroups in the three clusters (**Figure 3J**). These findings suggest that our risk score model based on the prognostic genes of the ASIG subgroup can well predict the prognosis of osteosarcoma patients and distinguish differences in the TME.

We further validated the risk score model with two validation cohorts. Using the abovementioned risk score calculation, the patients with osteosarcoma in the GEO dataset GSE21257 were split into two different risk score groups. The associations among risk score, patient survival rate, and prognostic factor expression in different groups were also investigated (**Supplementary Figures S1B, E**). The survival study revealed that the high-risk group had a worse prognosis (p = 0.0099) (**Supplementary Figure S1C**). According to the ROC analysis (**Supplementary Figure S1D**), the AUC values of the risk scores at 1, 3 and 5 years were 0.776, 0.699 and 0.631, respectively. We screened a sample of 56 individuals from TCGA-SARC containing connective tissue from the extremities, trunk, and pelvis, and bone tissue because osteosarcoma occurs in the connective tissue of the extremities and trunk (**Supplementary Table S1**). The patients were split into high- and low-risk groups according to the risk score. The overall survival of the high-risk group was poor (p = 0.027) (**Supplementary Figure S1F**), and the AUC analysis showed that the risk score had good predictive value (**Supplementary Figure S1G**).

### Development and calibration of columnar charts integrating clinical information and risk scores

**Figure 4:**
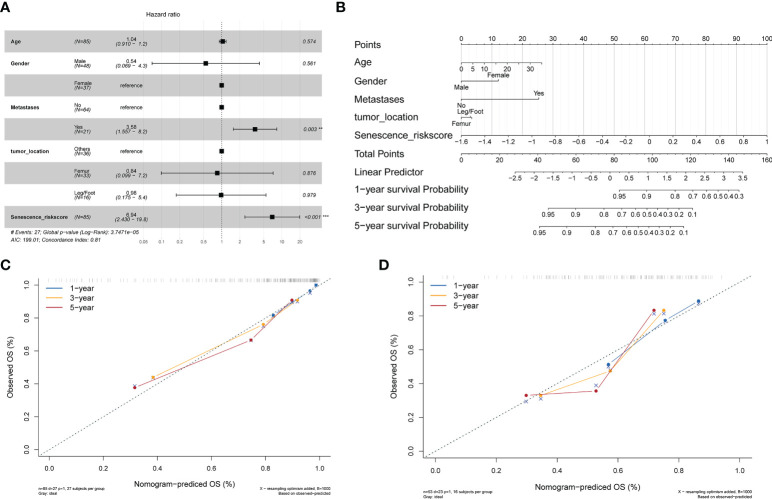
Development of a nomogram integrating clinical information. **(A)** Multivariate Cox analysis integrating clinical information and the risk score. **(B)** Nomogram for evaluating the 1- to 5-year overall survival of osteosarcoma patients. **(C)** Nomogram calibration for the training cohort (TARGET-OS) over 1–5 years. **(D)** Nomogram calibration for the verification cohort (GSE21257) over 1–5 years.

We combined the patients’ clinical characteristics to create line graphs and more accurately estimate the patients’ prognosis. First, we performed multivariate Cox regression analysis in the TARGET-OS cohort to determine that the risk score based on the four candidate genes is an independent risk factor relative to other clinical indicators (p< 0.001) (**Figure 4A**). The C-index reached 0.81 (95% CI, 0.772–0.848), demonstrating the high precision of the model. We then constructed a nomogram and scored the prognosis of patients according to their clinical indicators and risk scores (**Figure 4B**). In addition, we created an R Shiny app webpage (https://rshinyanalysisfigure.shinyapps.io/dynnomapp/) to enable interactive visualization for more convenient and precise patient prognosis prediction. The overall survival predicted by the nomogram was almost identical to the survival of the training (TARGET-OS) and validation (GSE21257) cohorts at 1, 3, and 5 years (**Figures 4C, D**). These results suggest that our developed comprehensive nomogram could precisely predict the prognosis of osteosarcoma patients.

### Association analysis between risk score and TIME and immune checkpoint blockade (ICB) response

**Figure 5:**
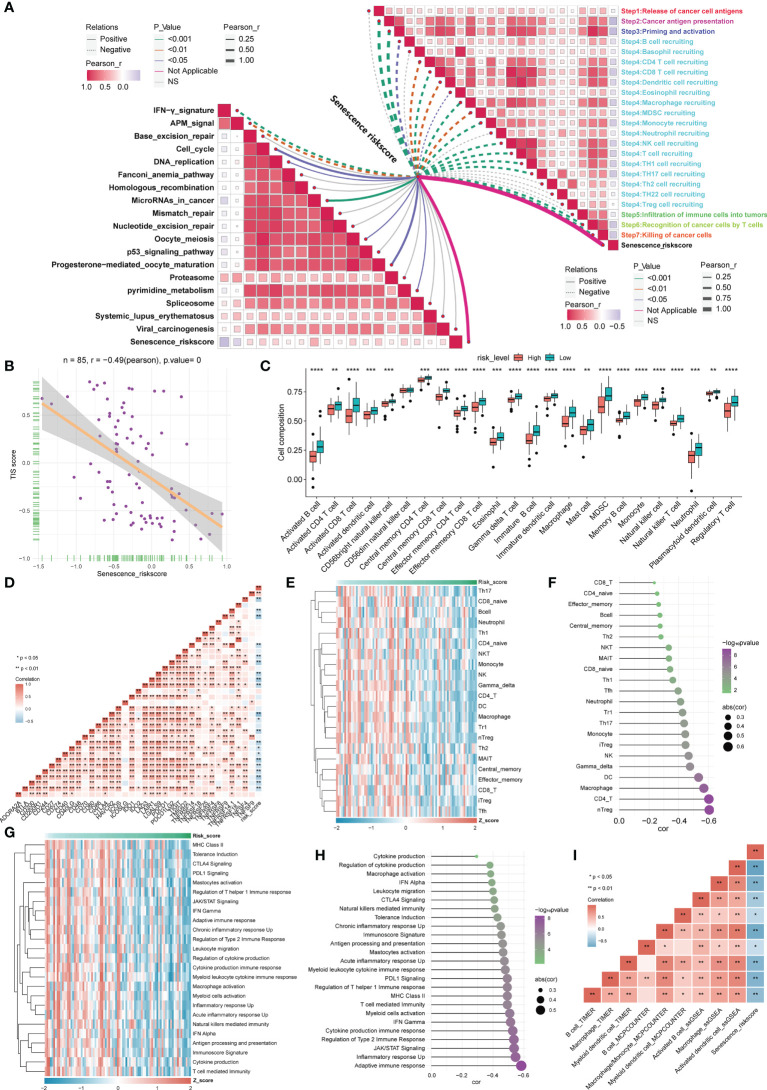
Correlation analysis between the risk score based on the ASIG subgroup and the TME and ICPs. **(A)** Correlation between the risk score constructed based on the four candidate genes and the tumor immunity cycle (right) and immunotherapy prediction pathways (left). **(B)** Correlation between T-cell score and risk score. **(C)** Distinctions in 28 immune cell infiltration levels between different risk score groups determined using the ssGSEA algorithm (*p< 0.05, **p< 0.01, ***p< 0.001, ****p< 0.0001). **(D)** Correlation analysis between the risk score constructed based on the four candidate genes and ICP. **(E, F)** Heatmap and correlation analysis between risk scores constructed based on the four candidate genes and tumor-infiltrating lymphocytes (TILs). **(G, H)** Heatmap of risk scores and innate immune pathways and correlation analysis. **(I)** The correlation between APC cell infiltration and risk score was assessed using three algorithms (TIMER, MCPCOUNTER, and ssGSEA).

Understanding the diversity of the TIME could effectively guide tumor immunotherapy. As a result, we examined the relationships between risk ratings based on ASIG clusters and the cancer immune cycle. Apparently, almost all seven processes, including cancer cell antigen release and presentation (steps 1 and 2), initiation and activation (step 3), and multiple immune cell recruitment (step 4), exhibited substantial inverse relationships with risk scores. Based on these results, we propose that high-risk patients with osteosarcoma may have tumors at an immunosuppressive state, whereas a low risk score for the inflammatory phenotype may indicate increased responsiveness to immune checkpoint blockade (ICB) therapy. The risk score was inversely related to the enrichment of some positive signals associated with immunotherapy [e.g., interferon (IFN) signals], but more predictive pathways mediating immunotherapy were positively related to risk scores, implying that immunotherapy may still be effective for high-risk patients (**Figure 5A**). The T-cell-associated inflammatory signature (TIS) score was also found to be strongly and inversely connected with the risk score (**Figure 5B**). Using the ssGSEA algorithm, the 28 immune cells were found to be lower in the high-risk group, indicating an immune “cold” phenotype, and higher in the low-risk group, indicating an immune “hot” phenotype. The risk score was inversely linked with most immune checkpoints (ICPs), as shown in **Figures 5C, D**, implying an immune escape mechanism and a better immunotherapeutic outcome for tumor cells in the low-risk group. This finding suggests that osteosarcoma patients in the low-risk group may still benefit from immunotherapy. In contrast, we assessed the relative abundance of 22 tumor-infiltrating immune cells (TIICs) (**Figures 5E, F**) in osteosarcoma patients in the high-risk group by considering the inverse correlation feature with the TIS score and further evaluated the correlation between innate immunity (**Figures 5G, H**) and risk score, which indicated that infiltrating immune cells in the high-risk group are associated with a lack of innate immune activation. We showed a significant inverse correlation between APC cell infiltration and the risk score using multiple algorithms (**Figure 5I**). Similar results were obtained with the validation cohort (**Supplementary Figure S2**). The above research results suggest that patients in the high-risk group may have more immunotherapy possibilities if a strategy to induce APCs in the TME to promote strong innate signaling could indeed help improve the cross-initiation of tumor antigen-specific CD8+ T cells by increasing chemokine production for effector T-cell trafficking. In contrast, the inflammatory profile of low-risk osteosarcoma patients may still be susceptible to immunotherapy.

### TME differences in cells associated with different risk scores

**Figure 6:**
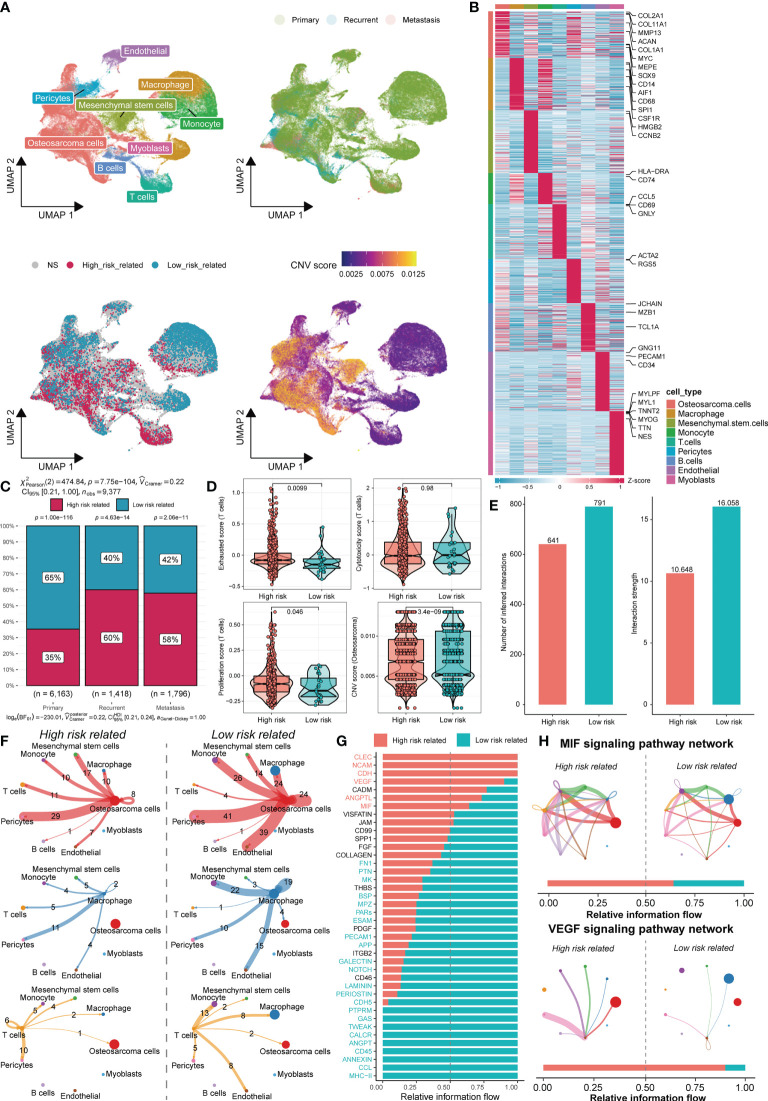
TME differences in cells associated with different risk scores. **(A)** UMAP analysis identified nine major cell types in osteosarcoma samples (top left), sample groupings involving primary, recurrent, and metastasis (top right), associated cells by SCISSOR risk groupings (bottom left), and CNV score inferred by the infercnv-related heatmap (bottom right). **(B)** The DEGs and marker genes for nine major cell types are shown. The colors in the top and side bars indicate specific cell clusters. **(C)** A bar graph depicts the proportion of cells in different sample groupings that are associated with the risk score. **(D)** Boxplots depict the exhaustion (top left), toxicity (top right), and proliferation (bottom left) scores of T cells in the two risk score groups. The difference in the CNV score between osteosarcoma cells is shown (bottom right). **(E)** Differences in the number of cells associated with different risk scores and the intensity of their cellular communication. **(F)** Circos plots depict putative ligand–receptor interactions between T cells from the high-risk (left) and low-risk (right) associated cell groups and other cell clusters. Branches connect pairs of interacting cell types and indicate the number of events in the graph. **(G)** In the inferred network, the overall information flow differences for the different risk-group-associated cells are shown. **(H)** Circos plots show the MIF signaling pathway and VEGF signaling pathway of related cells in different risk groups.

We next sought to clarify the cell types associated with different risk score phenotypes within the TME. Single-cell sequencing data of 11 osteosarcoma patients, including 7 primary patients, 2 recurrent patients, and 2 lung metastasis patients, were downloaded from GSE152048. After quality control and elimination of sample batches, the filtered cells were clustered and annotated as nine major cell clusters, including osteosarcoma cells, macrophages, monocytes, endothelial cells, pericytes, mesenchymal stem cells, T cells, B cells, and myoblasts (**Figures 6A, B**). We used the SCISSOR algorithm (16) developed by Sun et al. with a combination of sequencing data of TARGET-OS patients with risk grouping information of the corresponding patients to clarify the cells associated with different risk phenotypes (**Figure 6A**). Using infercnv software with immune cells as a reference, a CNV score was calculated for individual cells (**Figure 6A**). Not surprisingly, more high-risk-related cells were identified in patient samples with recurrence and metastasis (**Figure 6C**). In the high-risk group, higher exhaustion and cell proliferation scores were found for T cells. However, the T-cell cytotoxicity scores did not appear to be significantly different. This finding demonstrated that T cells in the TME among patients in the high-risk groups may still have some proliferative potential, but immune escape occurs due to T-cell exhaustion-related mechanisms. Furthermore, the CNV score of osteosarcoma cells was significantly higher in the high-risk group than in the low-risk group, implying that the high-risk patients had a higher degree of tumor cell malignancy in osteosarcoma (**Figure 6D**). We used CellChat to infer the overall intercellular interactions based on ligand receptor signaling to characterize the cell interactions between the high- and low-risk groups. Surprisingly, we found that the cells in the high-risk group generally had less intercellular interactions (**Figure 6E**). Although communication among tumor cells, macrophages, and T cells is important in the TME, changes in communication among these cells in the TIME may be more important (**Figure 6F**). Moreover, we found enrichment of low-risk-related cells to the highest antigen presentation signal, validating the inhibition of the antigen presentation-related signaling pathway of APC cells in our previous high-risk group at the cellular level. The high-risk group was enriched with the MIF signaling pathway, the CLEC signaling pathway, which was previously reported to be associated with osteosarcoma growth and metastasis, and the vascular endothelial growth factor (VEGF) signaling pathway, which promotes blood vessel growth (**Figures 6G, H**). Overall, these results confirm the association of osteosarcoma immune activity and the tumor growth profile with risk-score-related groups based on DEGs related to senescence.

### Identification of potential therapeutic drugs for treatment of the high-risk-score group

**Figure 7:**
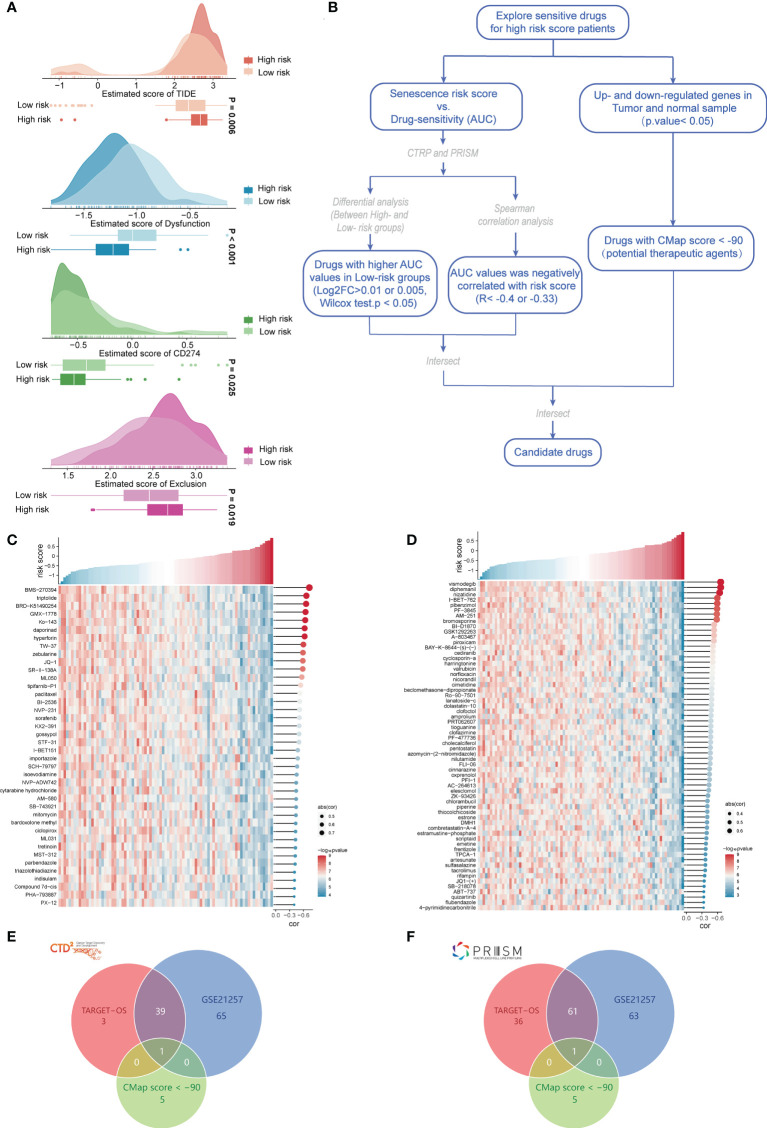
Evaluation of immunotherapy and screening of chemotherapeutic drugs. **(A)** Prediction of ICB treatment response of osteosarcoma based on the TIDE, dysfunction, CD274, and exclusion scores. **(B)** Flow chart of chemosensitivity prediction based on the osteosarcoma risk score. **(C)** Heatmap of the sensitivity of 40 chemotherapeutic drugs screened in the CTRP database. **(D)** Heatmap of the sensitivity of 62 chemotherapeutic drugs screened in the PRISM database. **(E)** Intersection with drugs with CMap scores< −90 in the CTRP database. **(F)** Intersection with drugs with CMap scores< −90 in the PRISM database.

We applied immunotherapy to predict the treatment effects and drug prediction based on risk scores. ICB therapy for cancer can have long-term clinical improvements, but only some patients respond to this treatment. Jiang et al. created TIDE algorithms (23) that integrate expression indicators of T-cell dysfunction and T-cell rejection to assess tumor immune evasion in the ability to forecast ICB response. Tumor cells with higher TIDE scores are more likely to trigger immunological escape, implying a reduced response rate to ICI therapy. Our outcomes indicated that the TIDE score was significantly lower in the low-risk group; that is, the low-risk group had greater immunotherapeutic potential, which supports our hypothesis. Unsurprisingly, the low-risk group exhibited greater T-cell dysfunction, whereas the high-risk group had higher T-cell rejection, which implies the existence of two distinct T-cell functional status phenotypes in the different risk groups, T-cell dysfunction and T-cell rejection. Based on the CD274 score, the low-risk group might have more options for PD-L1 immunotherapy (**Figure 7A**). For the high-risk group, we constructed a drug response prediction model using the PRISM and CTRP datasets. Hundreds of CCLs can be found in these two databases, which include expression and drug sensitivity information. Therefore, we built a drug response prediction model using these two databases, which include samples from hematopoietic, lymphoid tissue and cell lines, and removed compounds containing NA records. Finally, we used 680 CCLs (including 354 compounds) and 480 CCLs (including 1,285 compounds) from the CTRP and PRISM datasets, respectively, for subsequent analysis. Following the flow chart, we performed drug sensitivity prediction with TARGET-OS and GSE21257 and performed analyses using CTRP and PRISM, respectively (**Figure 7B**). Drug response analyses were performed for the different risk groups, and differences were assessed to identify compounds in the high-risk category with lower AUC estimations (CTRP: log2FC > 0.01; PRISM: log2FC > 0.005). We then performed Spearman correlation analysis of the AUC values with risk scores and selected derived compounds that exhibited a negative correlation with risk scores (CTRP: R< −0.4; PRISM: R< −0.33). Using TARGET-OS and GSE21257 with the CTRP dataset, we obtained 40 compounds, and using PRISM, we obtained 62 compounds, all of which had lower AUC estimates in the high-risk-score group and showed a significant inverse relation with the risk score (**Figures 7C, D**). Compounds with gene expression patterns that were opposite to the osteosarcoma expression patterns were identified through CMap analysis using cancer and paracancerous tissue data from GSE99671. We selected a subset of compounds with CMap scores< −90 and found one compound in each of the CTRP and PRISM datasets, including parbendazole and flubendazole, both of which had CMap scores< −95 (**Figures 7E, F**). In conclusion, evidence from multiple datasets and drug sensitivity estimates from multiple databases indicated that both parbendazole and flubendazole were potentially beneficial for osteosarcoma patients with high risk scores.

## Discussion

In diploid cells, cellular senescence, the occurrence of which was first described in the 1960s (24), is a persistent cell cycle arrest that limits their proliferative life span. This biological clock is triggered by the continual shortening of telomeres that occurs with each cell division and is a physiological reaction to avoid genomic instability and hence DNA damage build-up (25). Replicative senescence is the currently used term for this process. Senescent cells can accumulate during aging and at locations involved in age-related diseases, such as osteoarthritis (26) and atherosclerosis (27), which disrupts the normal physiology of tissues and leads to gradual functional degradation, even causing secondary damage to the body (28–30). Premature senescence is a term used to describe an accelerated senescence response in diploid cells that is not caused by telomere shortening (31). As soon as cells are exposed to certain insults, such as genotoxic stress or metabolic shock, as a result of culture conditions, the senescence response begins. Senescence can also be induced by oncogenic stress, which is caused by the overexpression of specific oncogenes or the deletion of TSGs in primary and tumor cells (32). Senescence has been observed *in vivo* in a number of malignancies and halts tumor development and progression. Senescence appears to be a strong anticancer mechanism due to its antiproliferative properties. The tumor-suppressive effect of senescence has cleared the way for cancer therapies that increase senescence, a procedure known as pro-senescence therapy for cancer. Despite their participation in a number of clinical illnesses, senescent cells are essential in physiological processes such as embryogenesis, tissue remodeling, and tissue repair (33). However, the involvement of senescence in the development, management, and prognosis of osteosarcoma remains unknown. We explored the expression of ASIGs in osteosarcoma tumor tissues and their relationships with osteosarcoma in the current investigation. First, a unique prognostic model incorporating four DEGs (EVI2B, AIM1, PRKACB, and TCEA3) belonging to ASIG subgroups was built and validated. Immune-related pathways were found to be involved in functional studies.

Although the processes driving tumor susceptibility to ASIGs have received much attention in recent years, the possible regulation of tumor immunity by ASIGs has remained a mystery. We found that many immune-related biological processes and pathways were prominent in GO analyses based on DEGs between different risk groups. It is reasonable to believe that ASIGs are associated with tumor immunity. Immune dysregulation is a common symptom of malignancies. In the TME, cellular senescence frequently triggers an immunological response (34), and immune cell infiltration promotes tumor progression (35). However, the significance of ARGs in immune cell modulation in osteosarcoma is uncertain. According to this study, osteosarcoma patients in the low-risk group may still benefit from immunotherapy. In contrast, we assessed the relative abundance of 22 TIICs in osteosarcoma patients in the high-risk group considering the inverse correlation feature with the TIS score and further evaluated the correlation between innate immunity and risk score, which indicated that infiltrating immune cells in the high-risk group are related to a lack of innate immune activation. We showed a significant inverse correlation between APC cell infiltration and the risk score using multiple algorithms. The above research results suggest that patients in the high-risk group may have more immunotherapy possibilities if a strategy to induce APCs in the TME to promote strong innate signaling could indeed help improve the cross-initiation of tumor antigen-specific CD8+ T cells by increasing chemokine production for effector T-cell trafficking. In contrast, the inflammatory profile of low-risk osteosarcoma patients may still be susceptible to immunotherapy.

Tumor cells, inflammatory cells, immune cells, mesenchymal stem cells, endothelial cells, and tumor-associated fibroblasts comprise the TME, which plays a different function in the proliferation, metastasis, and treatment resistance of tumor cells. Immunosuppression has been discovered to be common in the TME due to the lack of antigens in tumor cells and the lack of immunosuppression produced by immune system suppressive signaling pathways such as PD-1/PD-L1 and cytotoxic T-lymphocyte-associated antigen-4 (CTLA-4) (36). In addition, tumor cells alter infiltrating immune cells by secreting and releasing immunosuppressive substances such as transforming growth factor, interleukin-2, interleukin-10, and vascular endothelial growth factor into the microenvironment, which impede their antitumor activities (37). Furthermore, aberrant metabolic patterns amplify the immunosuppressive effects of the TME (38). Previous research has found that aging can cause comparable immune evasion. BRAFV600E mutant melanocytic nevi, for example, undergo senescence but are immune suppressed, which allows their accumulation during aging in humans (39). Additionally, when premalignant hepatocytes are not removed by the immune system after NrasG12 V-induced senescence, immature suppressive myeloid cells can be recruited, which restricts NK-cell activity and encourages hepatocellular carcinoma (HCC) progression (40). In this study, we observed that the high-risk group had higher T-cell depletion scores, reduced intercellular ligand–receptor communication, and weaker cellular antigen-presentation signaling using single-cell data analysis. As a result, we believe that aging-related genes reduce tumor immunity by boosting T-cell depletion and lowering cellular antigen presentation signals.

This study has several limitations. First, we built and evaluated our prognostic model using retrospective data from public databases. To confirm its clinical value, more prospective real-world evidence is needed. Second, the intrinsic shortcoming of constructing a prognostic model based solely on a single signature is inescapable because many significant prognostic genes in osteosarcoma may have been ignored. Third, in this study, cells associated with high and low risks were obtained through the combined analysis of RNA transcriptome data and single-cell data. More sample data from different risk patients are still needed to verify the reliability of this analysis. Furthermore, the association between risk score and immune response, and the outcomes of drug sensitivity prediction, needs to be studied experimentally.

In conclusion, aging-related prognostic genes can distinguish molecular subgroups of osteosarcoma. An innovative aging-related gene signature risk score can be utilized to predict prognosis. Moreover, the risk score was found to be linked to the TIME and was used to help navigate patient immunotherapy and chemotherapy in osteosarcoma.

## Data availability statement

The original contributions presented in the study are included in the article/**Supplementary Material**. Further inquiries can be directed to the corresponding authors.

## Ethics statement

Ethical review and approval was not required for the study on human participants in accordance with the local legislation and institutional requirements. Written informed consent from the patients/participants or patients/participants’ legal guardian/next of kin was not required to participate in this study in accordance with the national legislation and the institutional requirements.

## Author contributions

HZ, BS and XL designed and supervised the entire work. YGL, LW and HJ performed the experiments and contributed equally to this work. YGL and LW prepared and wrote the paper. YL, CZ, YFL and YK carried out the bioinformatics analysis. YGL, MH and ZL analyzed the data and prepared the figures. All authors contributed to the article and approved the submitted version.

## Funding

This study was supported by grants from the Science and Technology Development Fund of Tianjin Education Commission for Higher Education (2018KJ078).

## Conflict of interest

The authors declare that the research was conducted in the absence of any commercial or financial relationships that could be construed as a potential conflict of interest.

## Publisher’s note

All claims expressed in this article are solely those of the authors and do not necessarily represent those of their affiliated organizations, or those of the publisher, the editors and the reviewers. Any product that may be evaluated in this article, or claim that may be made by its manufacturer, is not guaranteed or endorsed by the publisher.

## Supplementary material

Supplementary Table 1 Clinical data for the entire cohort.

Supplementary Table 2 Demographic information and clinical characteristics of the training and validation cohorts.

Supplementary Table 3 Aging/senescence-induced genes.

Supplementary Table 4 Details of the cellular biomarkers.

Supplementary Table 5 Results of prognosis related genes in ASIGs in single-gene KM analysis and univariate Cox regression.

Supplementary Table 6 Gene list of immune cells used by ssGSEA algorithm.

Supplementary Table 7 List of genes used for CMap analysis and CMap score of all drugs.

Supplementary Figure 1 Independent survival evaluation of genes used to establish the risk score model and verification of the risk score using the verification cohort. **(A)** Survival curve of four candidate gene groups in the TARGET-OS cohort. **(B)** Risk score constructed from candidate genes, patient survival status, and expression heatmaps of the four candidate genes in the verification cohort (GSE21257). **(C)** Survival curve of the various risk score groups of the verification cohort (GSE21257). **(D)** ROC of the risk score constructed based on four candidate genes in the validation cohort (GSE21257). **(E)** Distribution of risk scores in the TCGA-SARC validation cohort and heatmap of candidate genes. **(F)** Survival curve of different risk score groups in the TCGA-SARC validation cohort. **(G)** ROC curve of the risk score constructed based on four candidate genes in the validation cohort (TCGA-SARC).

Supplementary Figure 2 The validation cohort (GSE21257) verified the association analysis between the risk score and immune microenvironment. **(A)** Correlation between risk scores in the validation cohort and tumour immune cycle (right) and immunotherapy prediction pathway (left). **(B)** Correlation between the risk score and T-cell score in the validation cohort. **(C)** The differences in the 28 immune cell infiltration levels between different risk score groups were evaluated in the validation cohort (* P< 0.05, ** P< 0.01, *** P< 0.001). **(D)** Correlation analysis between the risk score of the validation cohort and ICPs. **(E, F)** Heatmap and correlation between the risk score of the validation cohort and TILs. **(G, H)** Risk score, innate immune pathway heatmap and correlation analysis of the validation cohort. **(I)** The correlation between APC infiltration and the risk score of the validation cohort was evaluated using three algorithms (TIMER, MCPCOUNTER, and ssGSEA).
