# Supplementary material for: Identification and characterization of aging/senescence-induced genes in osteosarcoma and predicting clinical prognosis
Source: Front Immunol. 2022 Oct 5;13:997765. doi: 10.3389/fimmu.2022.997765 (PMC9579318; doi:10.3389/fimmu.2022.997765)
Supplement: Supplementary Table 2 — Demographic information and clinical characteristics of the training and validation cohorts. [file Table_2.docx]

Supplementary Table 2. Demographic information and clinical characteristics of the training and validation cohorts.

|  | Training cohort  (Target-OS, n = 85) | Validation cohort  (GSE21257, n = 53) | P-value |
| --- | --- | --- | --- |
|  | n/% | n/% |  |
| Age |  |  | 0.08 |
| <18 years | 66/77.6 | 34/64.2 |  |
| >=18 years | 19/22.4 | 19/35.8 |  |
| Sex |  |  | 0.37 |
| Female | 37/43.5 | 19/35.8 |  |
| Male | 48/56.5 | 34/64.2 |  |
| Survival status |  |  | 0.17 |
| Alive | 58/68.2 | 30/56.6 |  |
| Dead | 27/31.7 | 23/43.4 |  |
| Tumor location |  |  | 0.54 |
| Femur | 38/44.7 | 27/50.9 |  |
| Tibia | 21/24.7 | 15/28.3 |  |
| Fibula | 8/9.4 | 2/3.8 |  |
| Others | 18/21.2 | 9/17.0 |  |
