# Supplementary material for: Identification and characterization of aging/senescence-induced genes in osteosarcoma and predicting clinical prognosis
Source: Front Immunol. 2022 Oct 5;13:997765. doi: 10.3389/fimmu.2022.997765 (PMC9579318; doi:10.3389/fimmu.2022.997765)
Supplement: Supplementary Table 4 — Details of the cellular biomarkers. [file Table_4.docx]

**Supplementary Table 3.** The canonical markers for the 9 cell clusters in osteosarcoma tissues.

| cluster identity | Cell cluster | Marker genes | References |
| --- | --- | --- | --- |
| 1 | Osteosarcoma cells | COL1A1, MMP13, MEPE, MYC,COL2A1, ACAN, SOX9 | [1,2,3,4] |
| 2 | Macrophages | AIF1,CD68, CSF1R, SPI1,CD14, | [5,6] |
| 3 | Mesenchymal stem cells | NES, HMGB2 , CCNB2 | [1,7,8] |
| 4 | Monocyte | HLA-DRA, CD74 | [9] |
| 5 | T cells | CCL5, CD69, GNLY | [10,11] |
| 6 | Pericytes | ACTA2, RGS5 | [12] |
| 7 | B cells | JCHAIN, MZB1, TCL1A, MS4A1, CD19 | [12,13,14,15] |
| 8 | Endothelial cells | CD34, PECAM1, GNG11 | [16,17] |
| 9 | Myoblasts | MYOG, TNNT2, TTN , MYL1 , MYLPF | [18,19,20] |

This table is generated from the public literature cited.

**Supplementary References**

1. Baryawno N, Przybylski D, Kowalczyk M, Kfoury Y, Severe N, Gustafsson K, et al. A Cellular Taxonomy of the Bone Marrow Stroma in Homeostasis and Leukemia. *Cell* (2019) 177(7):1915-32.e16. doi: 10.1016/j.cell.2019.04.040.

2. Elsafadi M, Manikandan M, Atteya M, Hashmi J, Iqbal Z, Aldahmash A, et al. Characterization of Cellular and Molecular Heterogeneity of Bone Marrow Stromal Cells. *Stem cells international* (2016) 2016:9378081. doi: 10.1155/2016/9378081.

3. Mizoshiri N, Kishida T, Yamamoto K, Shirai T, Terauchi R, Tsuchida S, et al. Transduction of Oct6 or Oct9 Gene Concomitant with Myc Family Gene Induced Osteoblast-Like Phenotypic Conversion in Normal Human Fibroblasts. *Biochemical and biophysical research communications* (2015) 467(4):1110-6. doi: 10.1016/j.bbrc.2015.10.098.

4. Rowe P, Kumagai Y, Gutierrez G, Garrett I, Blacher R, Rosen D, et al. Mepe Has the Properties of an Osteoblastic Phosphatonin and Minhibin. *Bone* (2004) 34(2):303-19. doi: 10.1016/j.bone.2003.10.005.

5. Chen H, Ray-Gallet D, Zhang P, Hetherington C, Gonzalez D, Zhang D, et al. Pu.1 (Spi-1) Autoregulates Its Expression in Myeloid Cells. *Oncogene* (1995) 11(8):1549-60.

6. He J, Xu X, Francisco A, Ferrante A, Krakoff J. Markers of Adipose Tissue Macrophage Content Are Negatively Associated with Serum Hdl-C Concentrations. *Atherosclerosis* (2011) 215(1):243-6. doi: 10.1016/j.atherosclerosis.2010.12.018.

7. Dudakovic A, Camilleri E, Riester S, Lewallen E, Kvasha S, Chen X, et al. High-Resolution Molecular Validation of Self-Renewal and Spontaneous Differentiation in Clinical-Grade Adipose-Tissue Derived Human Mesenchymal Stem Cells. *Journal of cellular biochemistry* (2014) 115(10):1816-28. doi: 10.1002/jcb.24852.

8. Lee D, Taniguchi N, Sato K, Choijookhuu N, Hishikawa Y, Kataoka H, et al. Hmgb2 Is a Novel Adipogenic Factor That Regulates Ectopic Fat Infiltration in Skeletal Muscles. *Scientific reports* (2018) 8(1):9601. doi: 10.1038/s41598-018-28023-7.

9. Young M, Mitchell T, Vieira Braga F, Tran M, Stewart B, Ferdinand J, et al. Single-Cell Transcriptomes from Human Kidneys Reveal the Cellular Identity of Renal Tumors. *Science (New York, NY)* (2018) 361(6402):594-9. doi: 10.1126/science.aat1699.

10. Beura L, Wijeyesinghe S, Thompson E, Macchietto M, Rosato P, Pierson M, et al. T Cells in Nonlymphoid Tissues Give Rise to Lymph-Node-Resident Memory T Cells. *Immunity* (2018) 48(2):327-38.e5. doi: 10.1016/j.immuni.2018.01.015.

11. Zheng C, Zheng L, Yoo J, Guo H, Zhang Y, Guo X, et al. Landscape of Infiltrating T Cells in Liver Cancer Revealed by Single-Cell Sequencing. *Cell* (2017) 169(7):1342-56.e16. doi: 10.1016/j.cell.2017.05.035.

12. Kim N, Kim H, Lee K, Hong Y, Cho J, Choi J, et al. Single-Cell Rna Sequencing Demonstrates the Molecular and Cellular Reprogramming of Metastatic Lung Adenocarcinoma. *Nature communications* (2020) 11(1):2285. doi: 10.1038/s41467-020-16164-1.

13. Brinas F, Danger R, Brouard S. Tcl1a, B Cell Regulation and Tolerance in Renal Transplantation. *Cells* (2021) 10(6). doi: 10.3390/cells10061367.

14. Helmink B, Reddy S, Gao J, Zhang S, Basar R, Thakur R, et al. B Cells and Tertiary Lymphoid Structures Promote Immunotherapy Response. *Nature* (2020) 577(7791):549-55. doi: 10.1038/s41586-019-1922-8.

15. MacParland S, Liu J, Ma X, Innes B, Bartczak A, Gage B, et al. Single Cell Rna Sequencing of Human Liver Reveals Distinct Intrahepatic Macrophage Populations. *Nature communications* (2018) 9(1):4383. doi: 10.1038/s41467-018-06318-7.

16. Han X, Chen H, Huang D, Chen H, Fei L, Cheng C, et al. Mapping Human Pluripotent Stem Cell Differentiation Pathways Using High Throughput Single-Cell Rna-Sequencing. *Genome biology* (2018) 19(1):47. doi: 10.1186/s13059-018-1426-0.

17. Zhuo J, Fu W, Liu S. Correlation of Contrast-Enhanced Ultrasound with Two Distinct Types of Blood Vessels for the Assessment of Angiogenesis in Lewis Lung Carcinoma. *Ultraschall in der Medizin (Stuttgart, Germany : 1980)* (2014) 35(5):468-72. doi: 10.1055/s-0033-1356194.

18. Kong X, Wang X, Li M, Song W, Huang K, Zhang F, et al. Establishment of Myoblast Cell Line and Identification of Key Genes Regulating Myoblast Differentiation in a Marine Teleost, Sebastes Schlegelii. *Gene* (2021) 802:145869. doi: 10.1016/j.gene.2021.145869.

19. Niro C, Demignon J, Vincent S, Liu Y, Giordani J, Sgarioto N, et al. Six1 and Six4 Gene Expression Is Necessary to Activate the Fast-Type Muscle Gene Program in the Mouse Primary Myotome. *Developmental biology* (2010) 338(2):168-82. doi: 10.1016/j.ydbio.2009.11.031.

20. Zeng W, Jiang S, Kong X, El-Ali N, Ball A, Ma C, et al. Single-Nucleus Rna-Seq of Differentiating Human Myoblasts Reveals the Extent of Fate Heterogeneity. *Nucleic acids research* (2016) 44(21):e158. doi: 10.1093/nar/gkw739.
