# Supplementary figures and images for: Identification and characterization of aging/senescence-induced genes in osteosarcoma and predicting clinical prognosis
Source: Front Immunol. 2022 Oct 5;13:997765. doi: 10.3389/fimmu.2022.997765 (PMC9579318; doi:10.3389/fimmu.2022.997765)

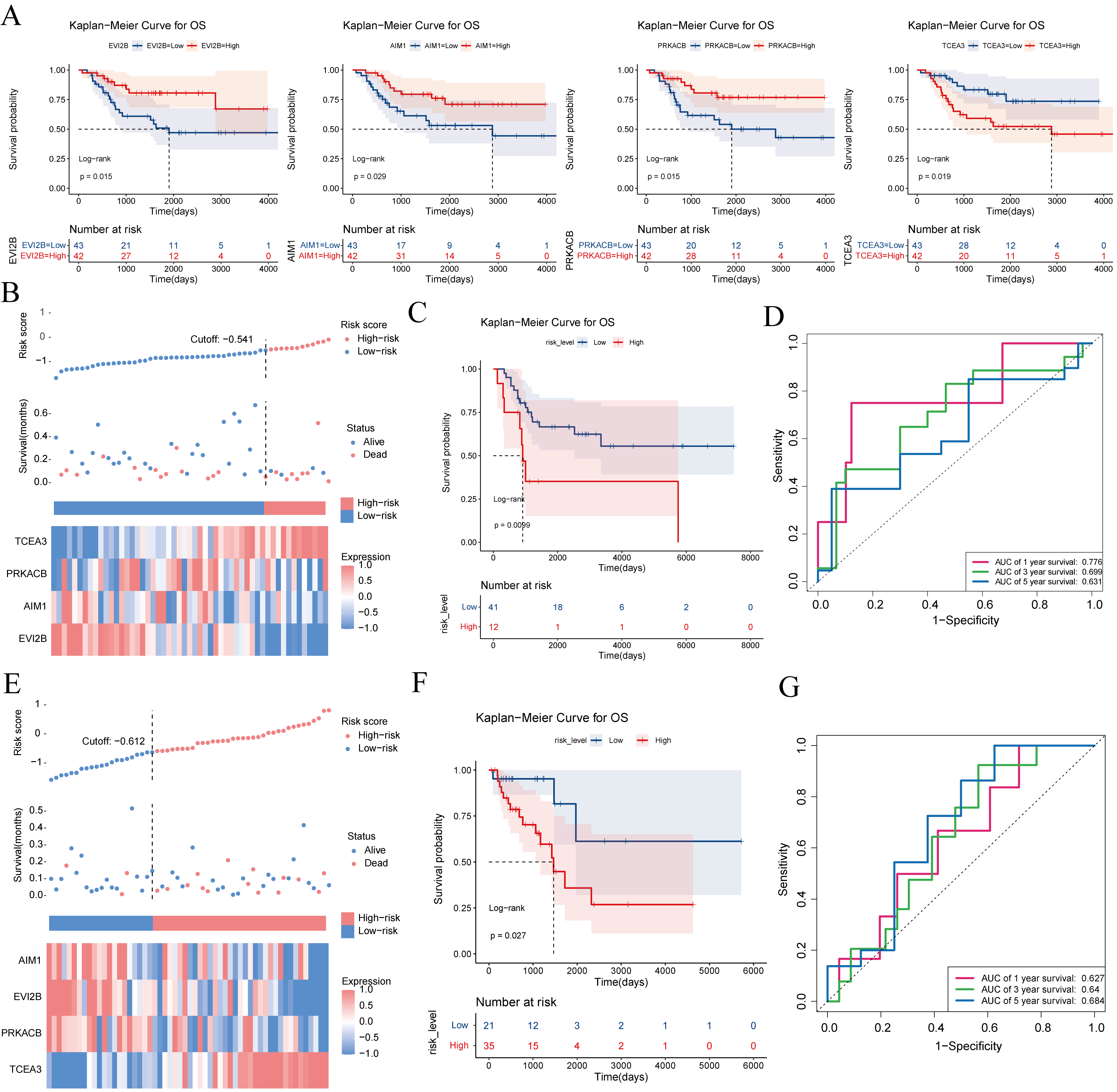

Supplement: Supplementary Figure 1 — Independent survival evaluation of genes used to establish the risk score model and verification of the risk score using the verification cohort. (A) Survival curve of four candidate gene groups in the TARGET-OS cohort. (B) Risk score constructed from candidate genes, patient survival status, and expression heatmaps of the four candidate genes in the verification cohort (GSE21257). (C) Survival curve of the various risk score groups of the verification cohort (GSE21257). (D) ROC of the risk score constructed based on four candidate genes in the validation cohort (GSE21257). (E) Distribution of risk scores in the TCGA-SARC validation cohort and heatmap of candidate genes. (F) Survival curve of different risk score groups in the TCGA-SARC validation cohort. (G) ROC curve of the risk score constructed based on four candidate genes in the validation cohort (TCGA-SARC). [file Image_1.tif]

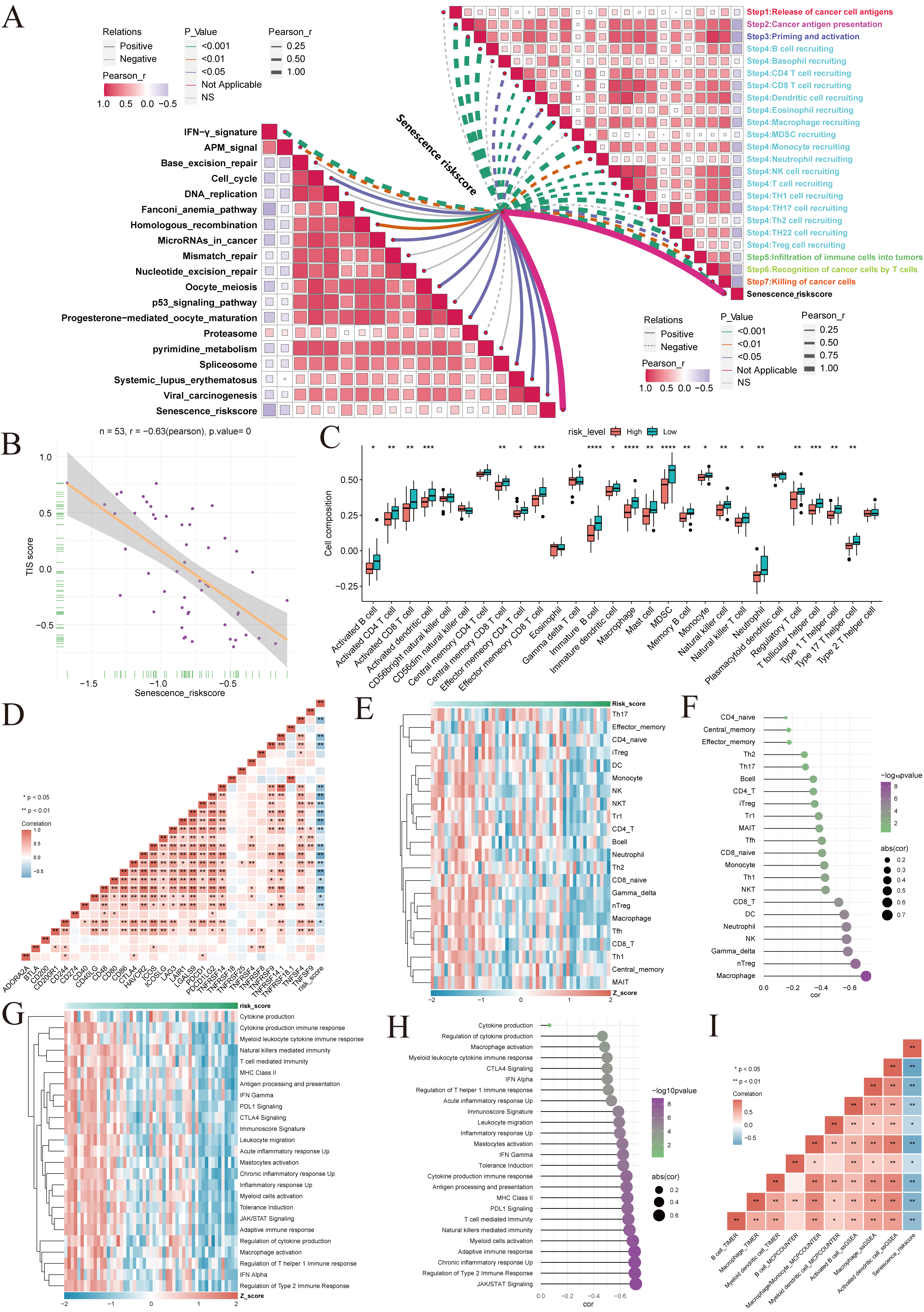

Supplement: Supplementary Figure 2 — The validation cohort (GSE21257) verified the association analysis between the risk score and immune microenvironment. (A) Correlation between risk scores in the validation cohort and tumour immune cycle (right) and immunotherapy prediction pathway (left). (B) Correlation between the risk score and T-cell score in the validation cohort. (C) The differences in the 28 immune cell infiltration levels between different risk score groups were evaluated in the validation cohort (* P< 0.05, ** P< 0.01, *** P< 0.001). (D) Correlation analysis between the risk score of the validation cohort and ICPs. (E, F) Heatmap and correlation between the risk score of the validation cohort and TILs. (G, H) Risk score, innate immune pathway heatmap and correlation analysis of the validation cohort. (I) The correlation between APC infiltration and the risk score of the validation cohort was evaluated using three algorithms (TIMER, MCPCOUNTER, and ssGSEA). [file Image_2.tif]
